# Editorial: The uterus immune microenvironment features in physiological and pathological conditions

**DOI:** 10.3389/fimmu.2026.1939464

**Published:** 2026-09-15

**Authors:** Md Zahidul I. Pranjol, Dmitry A. Zinovkin, Anna S. Portyanko, Hongbo Wang

**Affiliations:** 1 School of Life Sciences, Faculty of Science, Engineering and Medicine, University of Sussex, Brighton, United Kingdom; 2 Department of Pathology, Gomel State Medical University, Gomel, Belarus; 3 National Molecular Genetics Laboratory of Cancer Research, N.N. Alexandrov National Cancer Center of Belarus, Minsk, Belarus; 4 Department of Obstetrics and Gynecology, Union Hospital, Tongji Medical College, Huazhong University of Science and Technology, Wuhan, China

**Keywords:** cytokine networks in uterine homeostasis, decidual macrophages; immune tolerance in pregnancy, endometriosis-associated immune dysfunction, uterine cancer immune microenvironment, uterine natural killer (NK) cells

The uterus has spent too long being described as a passive vessel. For much of the history of reproductive biology it was treated as little more than a muscular chamber that holds a pregnancy and sheds its lining on a monthly schedule. That description is no longer tenable. The endometrium, decidua, myometrium and cervix together constitute one of the most restless immune environments in the human body: a tissue that remodels itself with every cycle, tolerates a semi-allogeneic fetus for nine months, heals without scarring after delivery, and, when this coordination breaks down, gives rise to some of the most common and stubborn disorders in women’s health. The twenty contributions to this Research Topic take that premise as their starting point. Collectively they shift the conversation away from simply cataloguing which immune cells reside in the uterus toward a harder and more useful set of questions: what those cells are doing, how they are instructed by their tissue neighbours, and how we might read their signals for diagnosis and therapy. The articles span original research, reviews and a clinical cohort, and they range across the reproductive life course, from the implanting embryo to the postmenopausal pelvic floor and the cancerous endometrium. Rather than survey each in turn, we have grouped them by the biological question they answer, because the most interesting patterns emerge when studies of very different diseases are set side by side and made to speak to one another.

## Building and un-building the healthy uterine niche

A recurring lesson from this collection is that uterine immunity is inseparable from tissue architecture. Wang et al. make the point vividly, combining single-cell and high-resolution spatial transcriptomics of the postmenopausal vaginal wall to show that estrogen does not simply switch tissue on. Instead it drives the selective expansion of HAS1+ fibroblasts that gather around blood vessels with pericytes to form an organised perivascular reparative niche. The finding reframes hormone action as the assembly of a spatially defined multicellular structure rather than diffuse activation, and it offers a plausible reason why local estrogen helps some women with pelvic organ prolapse and not others. A similar theme of ordered remodeling runs through Waldmann et al., who track how the uterus rebuilds itself after birth. Their analysis of human and mouse postpartum tissue identifies the mesometrial triangle as a distinct compartment where CD206-negative macrophages, CD8+ T cells and myofibroblasts assemble around the site of placental detachment, an inflammatory reaction deliberately walled off by a collagen capsule and tempered by IL-10 so that repair proceeds without fibrosis. Both papers argue that restraint, not just activation, is what healthy uterine immunity is made of.

Two further studies show how that physiology can be monitored. Chueh et al. followed cervical mucus across pregnancy in 133 women and found that the chemokines IP-10 and MIP-1β fall by roughly half from early to late gestation and track with mucus pH, pointing to a coordinated and non-invasively sampleable readout of gestational immune adaptation. Avalos et al. add a variable few would have predicted: fetal sex. Placental extracellular vesicles from male and female pregnancies reprogrammed maternal monocytes along different metabolic lines, with female-derived vesicles favouring a tolerogenic, lipid-fuelled phenotype and male-derived vesicles a more inflammatory one. That the sex of the fetus should tune the mother’s immunometabolism is the kind of result that should make us wary of treating pregnancy as a single, uniform immune state. It also carries a methodological warning that runs quietly through the whole collection: studies that pool male and female pregnancies, or that ignore where in the tissue a cell sits, may be averaging away the very signals they set out to find.

## The implantation window and early pregnancy tolerance

If the healthy uterus is defined by restraint, implantation is where that restraint is negotiated most delicately, and two mechanistic studies here converge on the idea that stromal cells, not immune cells, hold the script. Wang et al. show that decidual stromal cells secrete high-molecular-weight hyaluronic acid through HAS2, and that this matrix signal, acting on CD44, coaxes peripheral natural killer cells into tissue-resident, low-cytotoxicity dNK1-like cells via a Wnt and FOSL2 axis. In spontaneous abortion that instruction is lost and the NK compartment tilts toward cytotoxicity. Chen et al. tell a complementary story with the growth factor pleiotrophin, identifying it through shared analysis of recurrent implantation failure and recurrent pregnancy loss as a hub that drives decidualization and shapes the local immune milieu through IGF-2; silencing it in the mouse uterus impaired implantation, and IGF-2 supplementation rescued the defect. Both studies make a therapeutically attractive claim, that the endometrial stroma can be nudged toward tolerance rather than treated as inert scaffolding.

The clinical papers in this group are more sober about how far such biology has travelled toward the bedside. Rodriguez et al. sampled the embryo transfer catheter itself and found that a higher proportion of gamma-delta T cells associated with live birth while neutrophil enrichment tracked with failure, with modest discriminative power. It is a clever, low-cost idea, though the authors are appropriately careful that clinical utility is unproven in a cohort of 48. Wan et al. mine recurrent miscarriage transcriptomes for a less familiar form of cell death, paraptosis, and nominate PCNPP3 and ELOA as biomarkers linked to decidual NK cells and macrophages. These bioinformatics-led studies are hypothesis generators, and their value will stand or fall on independent validation, a caveat several authors in this issue commendably make on their own behalf. Taken together, the four papers sketch a satisfying arc from mechanism to marker, but they also expose the field’s central tension: the deepest biology comes from stromal and NK cell experiments in dishes and mice, while the human data remain observational and small. Closing that gap is the work that will decide whether any of these targets reaches a patient.

## When maternal-fetal tolerance fails

The pathological pregnancy papers share a common shape: an interface molecule goes missing or misbehaves, and immune balance collapses. Huang et al. report that the checkpoint ligand CD200 is downregulated in preeclamptic placenta, where it co-localises with CD68+ macrophages, and link its loss to systemic markers of organ injury, positioning it as both a candidate mechanism and a biomarker. Yin et al. cast a wider molecular net, using machine learning across preeclampsia and fetal growth restriction datasets to argue that placental glycosylation genes, among them B3GNT2 and ST6GAL1, are shared drivers of immune imbalance in both conditions. Glycosylation is an underexplored layer of maternal-fetal signalling, and its appearance here as a common thread linking two disorders that frequently coexist is a useful reframing rather than a mere gene list.

The two reviews in this section widen the lens further. Ma et al. make a persuasive case that obstetric antiphospholipid syndrome should be understood as more than a clotting disorder, foregrounding complement activation, neutrophil extracellular traps and decidual NK and macrophage dysfunction as the immune machinery that damages the placenta even in the absence of overt thrombosis. Sun et al. trace the many faces of CD147, a glycoprotein whose downregulation accompanies preeclampsia and growth restriction but whose overexpression fuels invasive trophoblastic disease. That duality is a caution to anyone hoping for simple therapeutic targets at the interface, where the same molecule can protect or destroy depending on context. What unites these four studies is a move away from viewing the failing placenta purely through the lens of blood flow and clotting, and toward an account in which immune cells, checkpoint ligands and sugar chains decide whether a pregnancy thrives. It is a more complicated picture, but a more honest one, and it opens targets that anticoagulation alone was never going to reach.

## Endometriosis and the ectopic immune microenvironment

Endometriosis attracts four contributions, and together they capture why the disease has resisted easy explanation. Xu and Du argue that immune evasion by ectopic lesions is not a single defect but an interlocking network of immune dysregulation, epigenetic silencing and metabolic reprogramming, with lactate from the hypoxic lesion doubling as a cofactor for the enzymes that rewrite chromatin. It is an ambitious synthesis, and its central implication, that single-target strategies will keep disappointing, deserves attention. Golinska et al. supply harder data through a network meta-analysis of nine datasets, spotlighting complement C3, coagulation and mast cells as lesion drivers and nominating JAK inhibitors as repurposing candidates. Where those two papers describe the problem, the other two probe ways out of it. Dong et al. show that ghrelin, acting through its receptor GHSR on endometrial stromal cells, suppresses TNF-α and IL-6 while raising IL-10, sketching an anti-inflammatory axis worth pursuing. Kalopedis et al. step outside the pelvis entirely to review how gut and reproductive tract dysbiosis may feed endometriosis-associated inflammation, weighing probiotics, faecal microbiota transplantation and short-chain fatty acid biomarkers with a welcome frankness about how thin the current evidence still is. Placed together, the quartet is a microcosm of endometriosis research as a whole: rich in plausible mechanisms and repurposing ideas, still short of the rigorous trials that would let clinicians choose between them.

## Infection, chronic inflammation and neoplasia

The final cluster returns immunology to the clinic, where infection and inflammation blur into disease. Yan et al. review chronic endometritis as a disorder of TLR and NLRP3 signalling, metabolic and epigenetic reprogramming, and a disturbed endometrial microbiome, a framing that helps explain why antibiotics alone so often disappoint. Liu et al. put that disappointment to the test in a retrospective cohort, finding that the timing of azithromycin, whether given immediately or after a delay, did not change cure rates or pregnancy outcomes in mild disease. It is a quietly valuable negative result in a field prone to over-treatment. Darville et al. dissect how Chlamydia infection of the human endometrium rewires miRNA and mRNA networks toward TGF-β signalling, epithelial-mesenchymal transition and regulatory T cell skewing, a combination that promotes scarring while dampening the very inflammation that might otherwise clear the pathogen. Finally, Chen et al. extend the microenvironment concept into malignancy, characterising calcyphosine as a probable tumour suppressor in endometrial carcinoma whose expression marks a differentiated, metabolically distinct cell state and correlates with richer immune crosstalk and better survival. The reach of this last paper, from a housekeeping calcium protein to survival curves and immune infiltration, is a reminder that the tools built to map the healthy uterine niche are exactly the ones now being turned on its cancers.

## Common threads and unfinished business

Several currents run through these twenty papers and deserve to be pulled harder. The first is spatial and single-cell resolution, which recurs from the postmenopausal vaginal wall to the postpartum myometrium and is quietly redrawing our maps of where uterine immunity actually happens. The second is the marriage of immunity and metabolism, visible in fetal-sex-specific monocyte programming, in lactate acting as an epigenetic signal, and in ghrelin’s rebalancing of cytokines. The third is the microbiome, which surfaces at the endometrial, cervicovaginal and gut levels as suspect, sensor and potential therapy at once. The fourth, and more sobering, is the distance between discovery and clinic: many of the biomarkers assembled here are elegant on paper yet tested in tens rather than thousands of patients, and the honest caveats their authors attach are as instructive as the findings themselves. If this collection carries a single message, it is that the uterine immune microenvironment is best understood as a set of tissue-embedded conversations rather than a roster of cells, and that learning to read, and eventually to rewrite, those conversations is where much of the next decade of work lies. We thank the authors and reviewers whose care made this Research Topic possible, and hope readers find in it as much provocation as answer.

